# Associations of C-Reactive Protein to Indices of Vascular Health and the Influence of Serum 25(OH)D Status in Healthy Adults

**DOI:** 10.1155/2012/475975

**Published:** 2012-09-16

**Authors:** Ambika P. Ashraf, Gordon Fisher, Jessica Alvarez, Tanja Dudenbostel, David A. Calhoun, Alexander J. Szalai, Barbara A. Gower

**Affiliations:** ^1^Division of Pediatric Endocrinology and Metabolism, Department of Pediatrics, The Children's Hospital, University of Alabama at Birmingham, Birmingham, AL 35233, USA; ^2^Department of Nutrition Sciences, University of Alabama at Birmingham, Birmingham, AL 35233, USA; ^3^Vascular Biology and Hypertension Program, University of Alabama at Birmingham, Birmingham, AL 35233, USA; ^4^Division of Clinical Immunology and Rheumatology, Department of Medicine, University of Alabama at Birmingham, Birmingham, AL 35233, USA

## Abstract

Elevated serum high-sensitivity C-reactive protein (hs-CRP) and low serum 25-hydroxyvitamin D [25(OH)D] are associated with increased cardiovascular disease (CVD) risk. Ethnic differences in serum hs-CRP and 25(OH)D concentrations and CVD are known. Objectives: to investigate the ethnic differences in hs-CRP concentrations, to assess the influence of 25(OH)D on these ethnic differences and to examine the influence of 25(OH)D on association between hs-CRP and cardiovascular health indices. Subjects: 62 healthy adults [26 African Americans (AA), 26 European Americans (EA), and 10 Hispanic Americans (HA)], ages 18–55 years. Serum hs-CRP and 25(OH)D as well as pulse wave velocity (PWV), augmentation index (AIx), and flow-mediated dilatation (FMD) were measured. hs-CRP was inversely associated with 25(OH)D (*r* = −0.25, *P* = 0.049), and hs-CRP was positively associated with PWV (*r* = 0.29, *P* = 0.04). The association of hs-CRP with PWV attenuated after adjustment for 25(OH)D (*P* = 0.15). hs-CRP was higher in AA compared to EA (*P* = 0.05); this differences was reduced by 32% after adjusting for serum 25(OH)D. Conclusion: eventhough the inverse association between serum 25(OH)D and CRP does not infer causality, lower serum 25(OH)D may increase risk for inflammation and endothelial dysfunction. The lower 25(OH)D in AA may predispose to greater inflammation and associated vascular dysfunction.

## 1. Introduction

Chronic inflammatory stress, endothelial dysfunction, and arterial stiffness are key underlying factors in the pathogenesis of atherosclerosis [1, 2]. C-reactive protein (CRP), an acute phase protein synthesized primarily in the liver, is an established biomarker of inflammation, is known to alter endothelial function via increased nitric oxide production [3] and its serum levels are positively associated with the development of cardiovascular disease (CVD) [4–6]. High-sensitivity C-reactive protein (hs-CRP) concentration is also associated with pulse wave velocity (PWV), the gold standard for assessing arterial stiffness, suggesting that inflammation is linked to arterial stiffness [7–9].

There is evidence that African Americans (AA) and Hispanic Americans (HA) have a greater risk of complications from CVD compared with European Americans (EA) [10, 11]. However to date, results vary as to whether or not inflammation differs between AA and EA. Khera et al. found higher hs-CRP concentrations among AA men and women between ages 30–65 years as compared to EA [12], whereas we have previously shown no differences in hs-CRP concentrations between AA and EA premenopausal women [13]. Additionally, AA have been shown to have greater arterial stiffness and endothelial dysfunction compared to EA [14–16]. Endothelial dysfunction and arterial stiffness are early and integral components of atherosclerosis and are novel biomarkers of CVD [17, 18]. Given the current magnitude of ethnic health disparities, it is paramount to identify and recognize race and ethnicity-related differences in cardiovascular risk factors. 

Vitamin D is hypothesized to have anti-inflammatory and cardioprotective properties and can potentially regulate proinflammatory cytokines [19, 20]. Serum concentration of 25-hydroxyvitamin D [25(OH)D], the indicator of vitamin D status, is associated with serum hs-CRP concentrations [21]. Several researchers have demonstrated a potential link between vitamin D status and noninvasive indices of arterial stiffness and brachial artery distensibility measured by flow-mediated dilatation (FMD) [22–24]. As AA are likely to have vitamin D deficiency [25], ethnic differences in vitamin D status may partially account for the ethnic differences in inflammatory biomarkers and vascular function. Although studies have examined the associations between hs-CRP, vascular resistance, and endothelial function indices [26, 27], the influence of 25(OH)D status on these outcomes is yet to be determined.

The primary objective of this study was to test the hypothesis that observed higher CRP in AA is due in part to their lower circulating vitamin D. A secondary objective was to determine whether vitamin D status influenced the associations between hs-CRP and cardiovascular risk markers (heart rate, brachial artery blood pressure (BP), central aortic BP, MAP, aortic PP, pulse wave velocity (PWV), augmentation index (AIx), and flow mediated dilatation (FMD)) in healthy nonobese adults.

## 2. Materials and Methods

Subjects were 62 adults, both sexes, age 18–55 years who were previously enrolled in 2 observational cohort studies: the VIVID study and the DIVA study (clinical trial registration numbers: NCT01041547, NCT01041365). The Institutional Review Board of the University of Alabama at Birmingham (UAB) approved both studies, and written informed consent was obtained before entry to the study. Ethnicity (European American—EA, African American—AA, or Hispanic American—HA) was self-reported. Exclusion criteria were diabetes, hypertension, or other conditions known to influence insulin sensitivity or vascular function; antihypertensive, glucose-controlling, or lipid-lowering medications, or other medications known to modify vascular function; vitamin D supplementation, smoking; BMI > 322 kg/m^2^, or lactose intolerance.

Testing was performed on 2 separate days in the same week. Blood samples, seated and supine systolic and diastolic blood pressure (SBP and DBP), and anthropometrics were obtained in the UAB Clinical Research Unit of the Center of Clinical and Translation Science (CCTS) and the Department of Nutrition Sciences after a 12 hr fast. Body composition (fat and lean mass) was assessed using dual-energy X-ray absorptiometry (iDXA, GE-LUNAR Radiation Corp., Madison, WI). 

During a second morning visit after an 8 hr fast, radial pulse wave analysis (PWA), carotid-femoral PWV, and FMD testing were conducted by a single physician in the Diabetes Research Training Center (DRTC) Human Physiology Core Cardiodynamic Laboratory. Brachial artery distensibility (FMD) was measured by brachial ultrasound imaging with a 7.5 MHz linear-array probe (Philips HP Agilent Sonos 5500, Andover, MA) according to standard guidelines [28]. Radial PWA and carotid-femoral PWV were performed using SphygmoCor applanation tonometry system (AtCor Medical, Sydney, Australia) as previously described [29]. Radial PWA was performed for assessment of augmentation index (AIx), AIx adjusted to a heart rate of 75 beat/min (AIx75), central aortic systolic blood pressure (aSBP) and central aortic diastolic blood pressure (aDBP).

All analyses were conducted in the Core Laboratory of the University of Alabama at Birmingham DRTC. hs-CRP was assayed with the Stanbio Sirrus using a turbidimetric procedure. Mean sensitivity is 0.50 mg/L and interassay CV is 8.9%. Serum 25(OH)D concentrations were assayed with a liquid chromatography-tandem mass spectrometry technique (Quest Diagnostics Nichols Institute, San Juan Capistrano, CA).

### 2.1. Statistical Analyses

Descriptive characteristics are reported as means (±SDs). The distributions of all variables were examined, and variables that deviated from a normal distribution were log10-transformed prior to statistical analyses. Between-race differences for each variable were determined using a one-way ANOVA. Bonferroni post hoc analyses were performed when statistically significant differences were observed. In order to further explore factors that contributed to observed differences between race, a univariate analysis was performed on variables that were statistically significant, using %fat, 25(OH)D, as covariates in the analyses. Hispanic subjects were not included in the race analysis due to their smaller number. To determine if subjects differed based on their vitamin D status, subjects were divided based on serum 25(OH)D ≥ 20 ng/mL and <20 ng/mL [30, 31]. Group differences according to serum 25(OH)D status for each variable were determined with 2-group *t-*tests. Pearson correlation analyses were used to investigate the relationships between hs-CRP, serum 25(OH)D, and vascular measures. Serum 25(OH)D and percent body fat were further examined as potential confounders in MLR analyses to investigate the relationships between hs-CRP and vascular outcomes. All analyses were performed using the statistical package for the social sciences (SPSS, version 19.0, Chicago, IL).

## 3. Results

**Figure 1:**
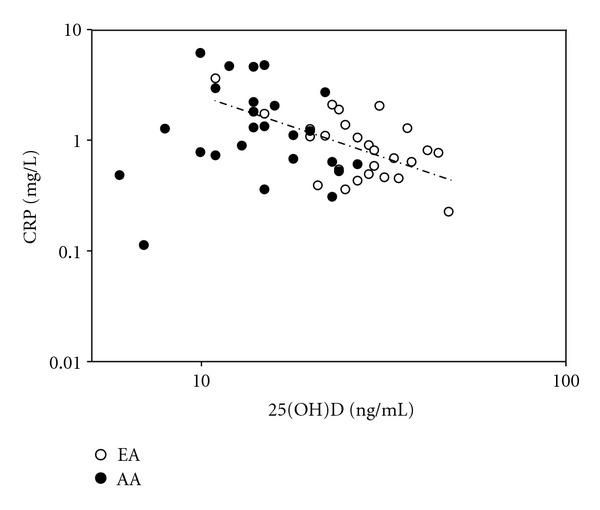
Associations of serum hs-CRP with 25(OH)D. hs-CRP was inversely associated with 25(OH)D (*r* = −0.25, *P* = 0.049) as a group. In EA *r* = −0.544, *P* = 0.004 and in AA, there was no relationship *r* = 0.015, *P* = 0.94.

**Figure 2:**
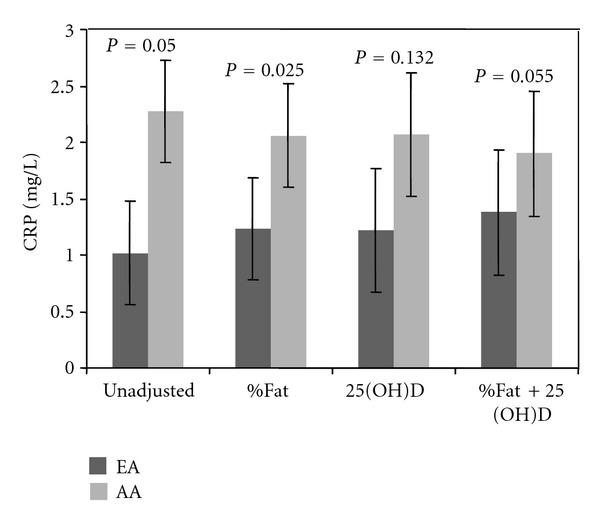
Ethnic differences in hs-CRP (a) unadjusted, (b) adjusted for percent body fat, (c) adjusted for 25(OH)D, and (d) further adjusted for percent body fat and 25(OH)D. After adjusting for serum concentration of 25(OH)D, there was a 32% reduction in hs-CRP difference between AA and EA.

A total of 62 subjects (26 EA, 26 AA, 10 HA) were included. Hispanic subjects were not included in the ethnic-specific analysis due to their limited number. AA had lower 25(OH)D (*P* = 0.001) and higher BMI (*P* = 0.03), percent body fat (*P* = 0.02), AIx75 (*P* = 0.001), PWV (*P* = 0.002), and CRP (*P* = 0.05) compared to EA—see Table 1. When EA and AA subjects combined, hs-CRP was inversely associated with 25(OH)D (*r* = −0.25, *P* = 0.049)—see Figure 1. The association of hs-CRP with25(OH)D persisted after adjusting for age, sex, and percent body fat, but was attenuated after adjusting for race (*P* = 0.069)—see Figure 1. After adjustment for 25(OH)D, the ethnic difference in CRP was reduced by 32% (*P* = 0.13)—see Figure 2. In subanalysis, hs-CRP was inversely associated with 25(OH)D in EA (*r* = −0.54, *P* = 0.004), and this persisted even after adjustment of age, sex, and percent body fat (*r* = −0.58, *P* = 0.002). hs-CRP was not associated with vitamin D status in AA. Due to the small sample size, we could not conduct analyses within the HA group. 

Serum 25(OH)D was associated with PWV after adjusting for race (*r* = −0.303; *P* = 0.034). hs-CRP was positively associated with PWV (*r* = 0.29, *P* = 0.04) and heart rate (*r* = 0.295, *P* = 0.02), but not with brachial or aortic SBP/ DBP, mean arterial pressure (MAP), aortic pulse pressure (PP), AIx75, or FMD. The association of hs-CRP with PWV attenuated after adjustment for BMI (*r* = 0.24, *P* = 0.09) and disappeared after adjustment for 25(OH)D concentrations (*r* = 0.20, *P* = 0.15) and after adjustment for race (*r* = 0.261; *P* = 0.07). The association with heart rate persisted after adjustment for percent fat and 25(OH)D.

When classified based on serum 25(OH)D concentrations (<20 ng/mL and >20 ng/mL), 27 subjects (43.5%) had serum 25(OH)D <20 ng/mL. Subjects with <20 ng/mL 25(OH)D had higher body weight (*P* = 0.002), BMI (*P* = 0.03), hs-CRP (*P* = 0.001), AIx75 (*P* = 0.001), and PWV (*P* = 0.015) as compared to individuals with serum 25(OH)D >20 ng/mL, when adjusting for age, race, %fat, and gender.

## 4. Discussion

 In the present study, we have shown that hs-CRP is higher in AA, and this ethnic difference is attenuated by adjustment for vitamin D status. Moreover, hs-CRP is positively associated with PWV (a measure of arterial stiffness) and vitamin D may influence this association. These observations suggest that relatively low circulating concentrations of 25(OH)D may increase risk for inflammation and endothelial dysfunction, even in healthy young adults.

Serum hs-CRP is an independent risk factor for development of hypertension, endothelial dysfunction, arterial stiffness, and atherosclerosis [5, 32, 33]. We confirmed that AA have poorer vascular function indices, lower 25(OH)D concentrations, and higher hs-CRP, as shown in other studies [16, 34]. AA subjects also reportedly have a greater prevalence of coronary heart disease and mortality rates [35]. Although ethnic differences in vascular function are well known, the pathophysiological basis for these differences remains elusive. It is conceivable that vitamin D deficiency may trigger acute phase-reactant production, equivalent to a low-grade systemic inflammation. After adjustment for percent fat and serum 25(OH)D, the difference in hs-CRP between AA and EA was diminished, which may have relevant clinical implications and partially explain these ethnic differences. 

In our cohort, hs-CRP was associated with serum 25(OH)D which is consistent with previous reports [36]. Nevertheless, in our data, serum 25(OH)D was strongly associated with hs-CRP only in EA and the association persisted even after adjusting for BMI and percent fat. One explanation for the absence of an association of 25(OH)D and hs-CRP in AA could be masked by their almost ubiquitous vitamin D deficiency. As most of the AA subjects had a 25(OH)D <20 ng/mL, a large number of subjects may be required to detect an association. In our sample, the EA were predominantly responsible for the observed relationship between hs-CRP and 25(OH)D. With a larger sample size of AA subject, a possible association may manifest. A noteworthy observation by Amer and Qayyum [36] in a large cohort of asymptomatic adults shows that there was reduction in CRP for each 10 ng/mL increase in serum 25(OH)D as long as serum 25(OH)D was ≤ 21 ng/mL, and CRP started increasing with increasing 25(OH)D concentrations above > 21 ng/mL. They have not reported ethnic-specific cutoffs for this finding. Although the implications of these observations are not clear, there could be different threshold levels in AA and EA above which raising the 25(OH)D concentrations may not be helpful in improving the hs-CRP.

In this study, we observed an association between CRP and PWV. It has been reported that PWV is an independent predictor of cardiovascular events [37]. We found that adjustment for serum 25(OH)D, attenuated the association between hs-CRP and PWV, suggesting that vitamin D status influences this relationship. Several reports have previously alluded to the fact that vitamin D can serve as an anti-inflammatory agent. Vitamin D3 supplementation was found to reduce the proinflammatory cytokine tumor necrosis factor *α* and enhance the anti-inflammatory interleukin 10, in a cohort of subjects with congestive heart failure. However, their CRP did not change [38]. Because of the inflammatory component of atherosclerosis and because elevated hs-CRP is reportedly associated with cardiovascular disease, it is critical to understand the factors responsible for elevated hs-CRP. Vitamin D supplementation may be an additive therapeutic option for conditions accompanied by increased inflammation and a subsequent decline in cardiovascular health.

Limitations of the study are cross-sectional nature and relatively smaller sample size which limits our ability to conclude causality based on observational associations. Controlled clinical trials are required to clarify whether vitamin D supplementation reduces ethnicity differences in hs-CRP and whether vitamin D supplementation influences the associations between hs-CRP and vascular health indices.

Strengths of the study are inclusion of subjects with body mass index <32 kg/m^2^, inclusion of DXA measure of body fat, and inclusion of young healthy adults—a population where preventive interventions could have a profound impact on health. Moreover, we were able to utilize direct accurate measures of arterial stiffness, endothelial function, and central BP parameters.

We conclude that ethnic differences in hs-CRP may be mediated by unequal vitamin D status. Likewise, hs-CRP is higher in subjects with vitamin D deficiency. Furthermore, serum 25(OH)D influences the association between hs-CRP and PWV, a direct measure of arterial stiffness.

## Figures and Tables

**Table 1:** Descriptive statistics and outcome variables by race in young adults.

Variable	EA (*N* = 26)	AA (*N* = 26)	HA (*N* = 10)	*P* value ^∗^ *P*
Age (years)	28 ± 9.9	32 ± 10.5	28 ± 5.9	*P* = 0.36
Females %	76.9%	84.6%	40%	*P* = **0.02**
BMI (kg/m^2^)	23.3 ± 3.1	25.6 ± 4	25.5 ± 3.7	*P* = 0.06 ^∗^ *P* = **0.03** ^a^
Waist circumference (cm)	74.7 ± 9.2	78.9 ± 10.6	83.9 ± 11	*P* = **0.05**
Percent body fat (%)	29.1 ± 6.9	34.2 ± 8.3	31.8 ± 8.5	*P* = 0.06 ^∗^ *P* = **0.02** ^b^
25(OH)D (ng/mL)	28.6 ± 8.8	15.3 ± 5.5	24.2 ± 5.3	*P* = **0.001** ^∗^ *P* = **0.001** ^c^
hs-CRP (mg/L)	1.01 ± 0.73	2.3 ± 3.2	1.04 ± 0.58	*P* = 0.09 ^∗^ *P* = **0.05** ^d^
Heart rate (beats/min)	64.9 ± 9.2	70.1 ± 12.3	68.4 ± 7.3	*P* = 0.20
Brachial SBP (mm Hg)	112.3 ± 13.8	111.2 ± 14.3	113.8 ± 11.2	*P* = 0.88
Brachial DBP (mm Hg)	68.4 ± 7.9	68.8 ± 9.1	71.3 ± 12.5	*P* = 0.7
Aortic SBP (mm Hg)	99.6 ± 9.6	102.9 ± 6.9	102.9 ± 11.8	*P* = 0.35
Aortic DBP (mm Hg)	70.4 ± 9.4	73.7 ± 6.7	77.1 ± 8.5	*P* = 0.090
MAP (mm Hg)	82.4 ± 8.7	83.7 ± 10.1	85.3 ± 10.5	*P* = 0.71
Aortic PP	29.2 ± 6.7	29.3 ± 4.7	25.8 ± 4.9	*P* = 0.25
FMD (%)	10.6 ± 3.9	8.8 ± 2.8	7.8 ± 2.2	*P* = **0.04** ^∗^ *P* = **0.07**
Aix at 75 (%)	0.74 ± 12.1	10.9 ± 8.6	2.2 ± 12.3	*P* = **0.005** ^∗^ *P* = **0.001** ^e^
PWV (m/s)	6.2 ± 0.7	7.1 ± 1.1	6.5 ± 0.70	*P* = **0.006** ^∗^ *P* = **0.002** ^f^

^∗∗^Bold face values denote significance *P* < 0.05. *P* = between 3 races.

^∗^ *P* ^a, b, c, d, e, f^ = super scripts are significant *P* values showing differences between AA and EA for post hoc analysis.

Continuous variables are expressed as mean ± SD, categorical variables are expressed as percentage.

Abbreviations: MAP: mean arterial pressure; Aortic PP: aortic pulse pressure; AIx75: augmentation index standardized to a heart rate of 75 beats/min; PWV: pulse wave velocity; FMD: flow-mediated dilation; SBP: systolic blood pressure; DBP: diastolic blood pressure.
